# Supplementary figures and images for: Active regulation of the epidermal growth factor receptor by the membrane bilayer
Source: eLife. 2026 Apr 14;14:RP108789. doi: 10.7554/eLife.108789 (PMC13078784; doi:10.7554/eLife.108789)

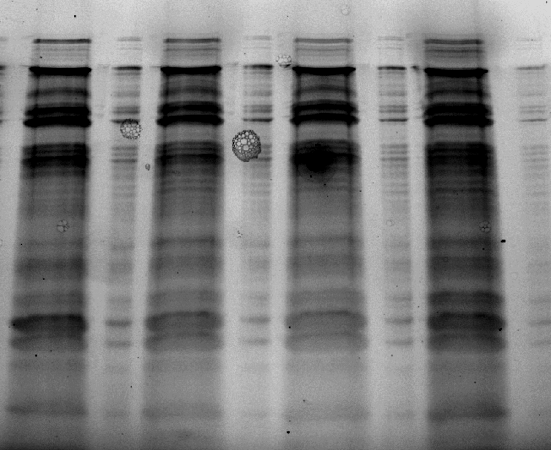

Supplement: Figure 1—figure supplement 2—source data 2. [file elife-108789-fig1-figsupp2-data2.zip › Figure 1 - figure supplement 2b - Stainfree gel.tif]

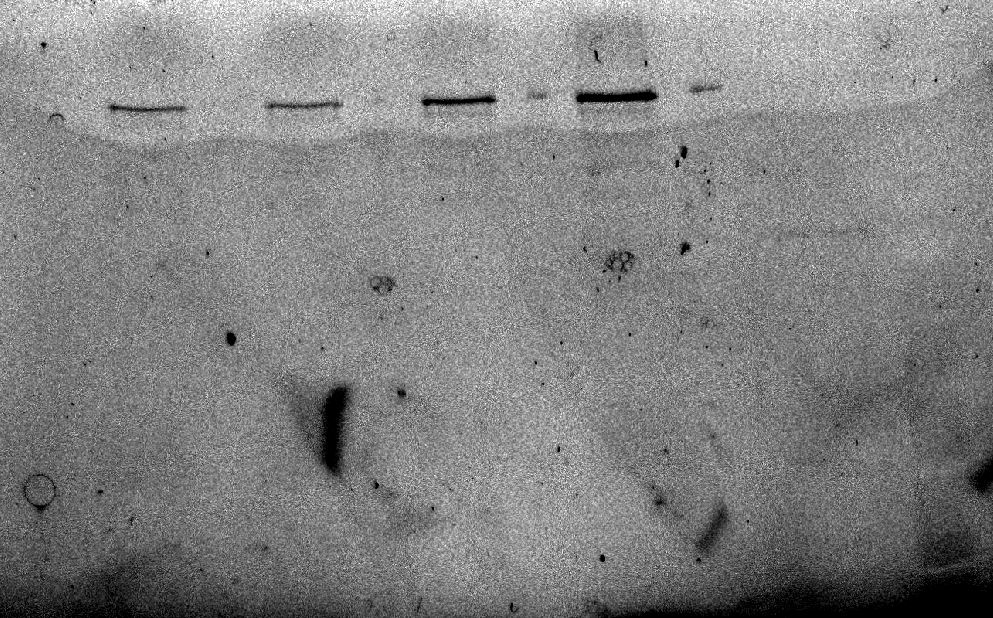

Supplement: Figure 1—figure supplement 2—source data 2. [file elife-108789-fig1-figsupp2-data2.zip › Figure 1 - figure supplement 2b - Fluorescence gel.tif]

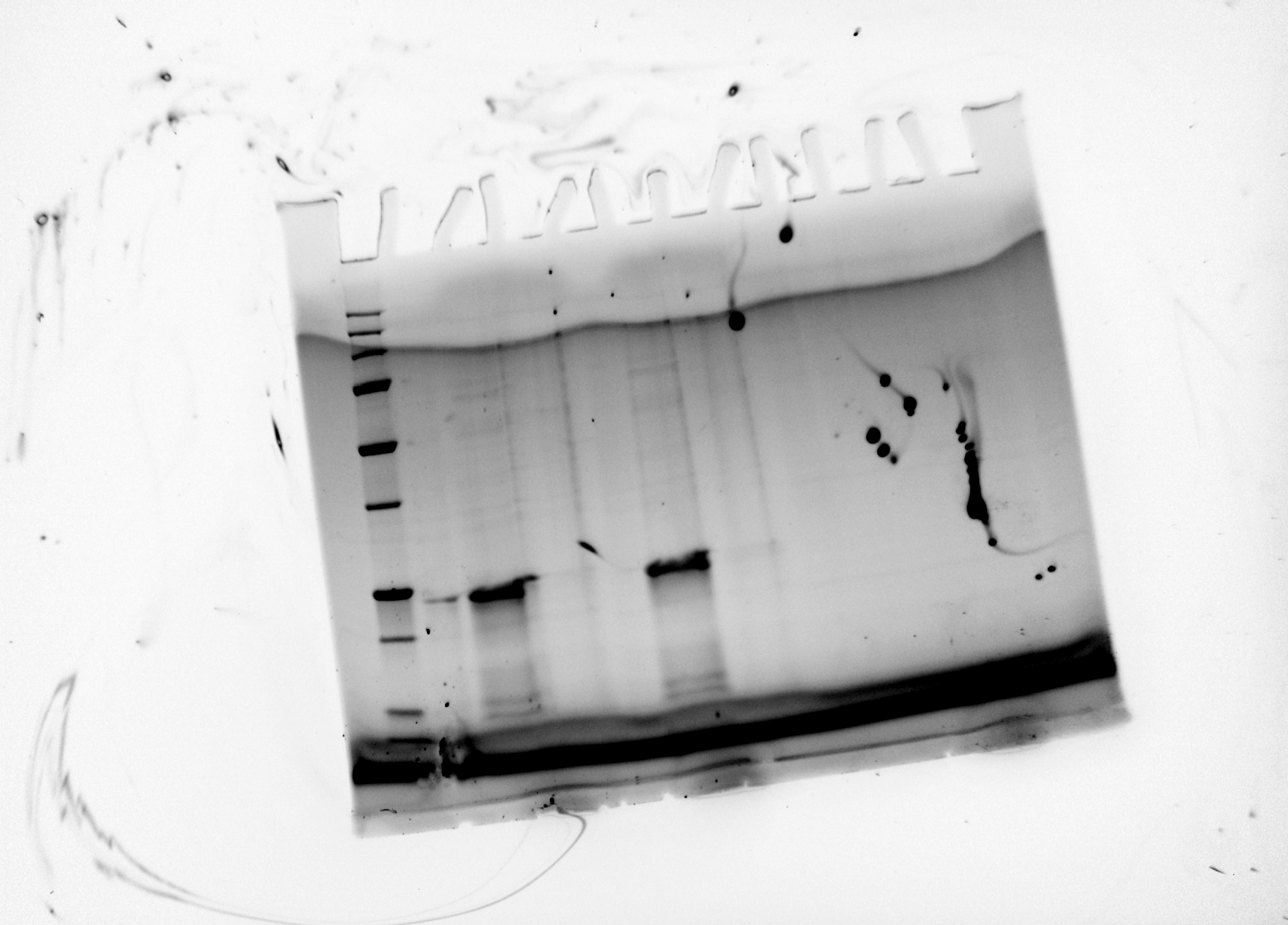

Supplement: Figure 1—figure supplement 2—source data 4. [file elife-108789-fig1-figsupp2-data4.zip › Figure1Supplement2c.tif]

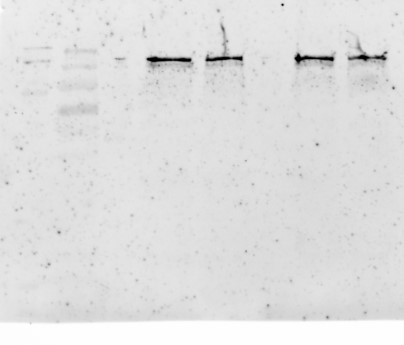

Supplement: Figure 1—figure supplement 2—source data 6. [file elife-108789-fig1-figsupp2-data6.zip › Fig1Supplement2d2.tif]

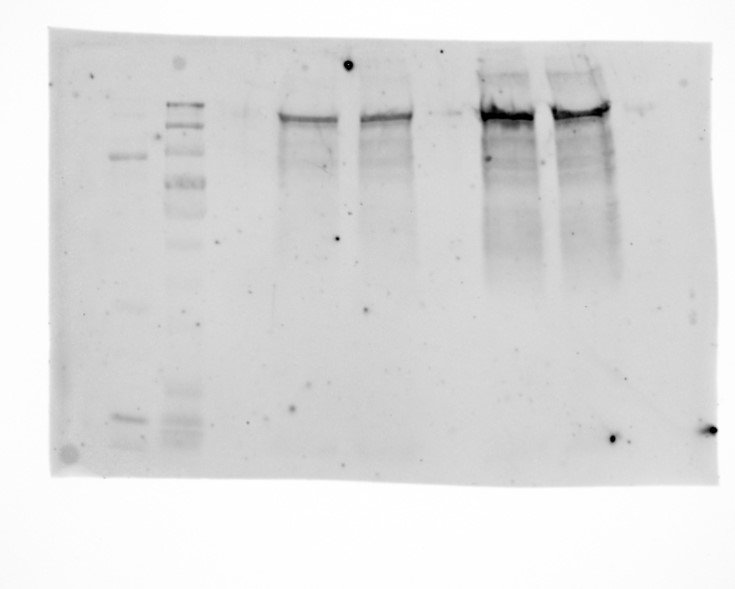

Supplement: Figure 1—figure supplement 2—source data 6. [file elife-108789-fig1-figsupp2-data6.zip › Fig1Supplement2d1.jpg]

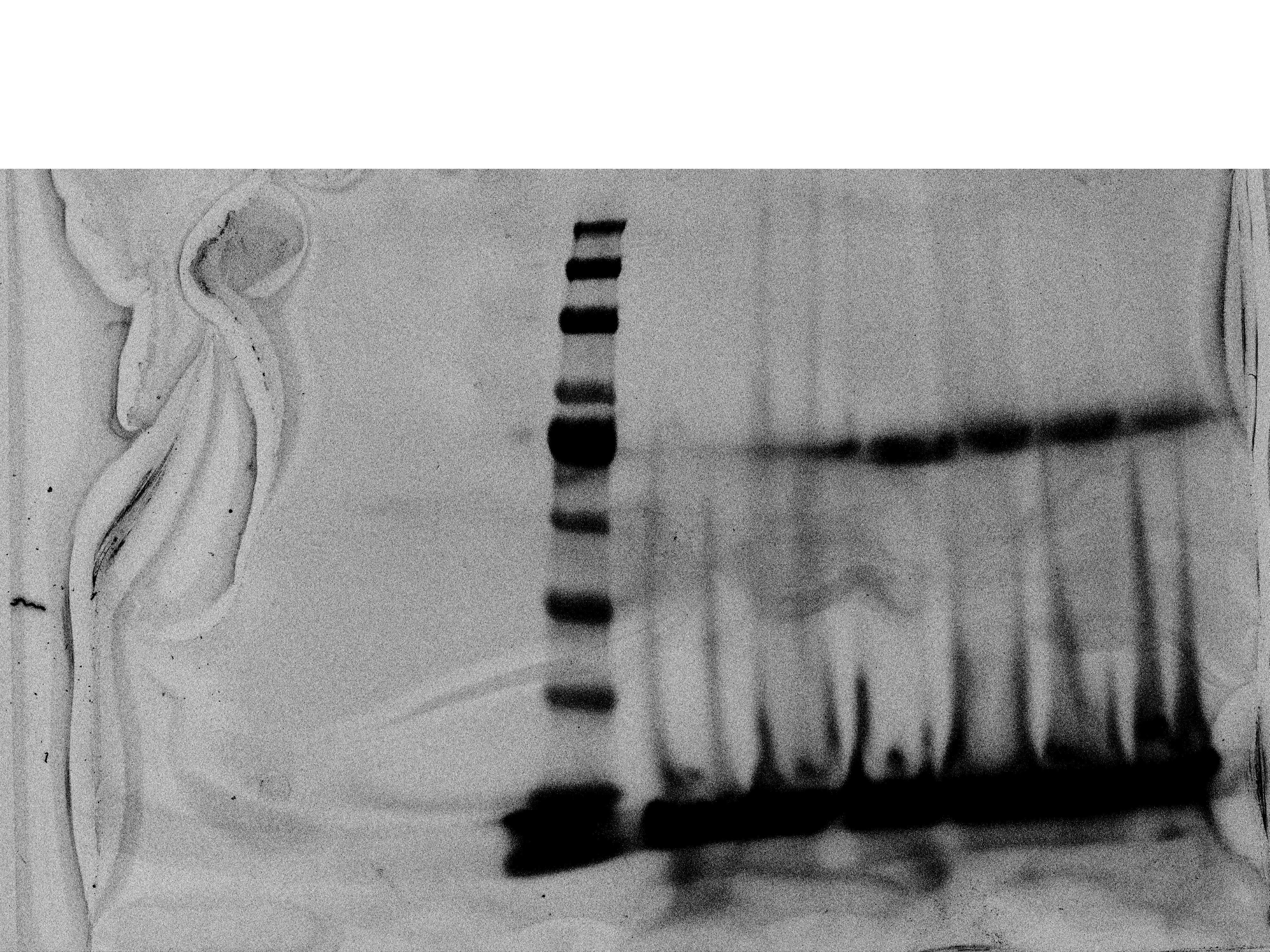

Supplement: Figure 1—figure supplement 3—source data 2. [file elife-108789-fig1-figsupp3-data2.zip › Figure 1 - figure supplement 3/Fig1Supplement3_19hours.jpg]

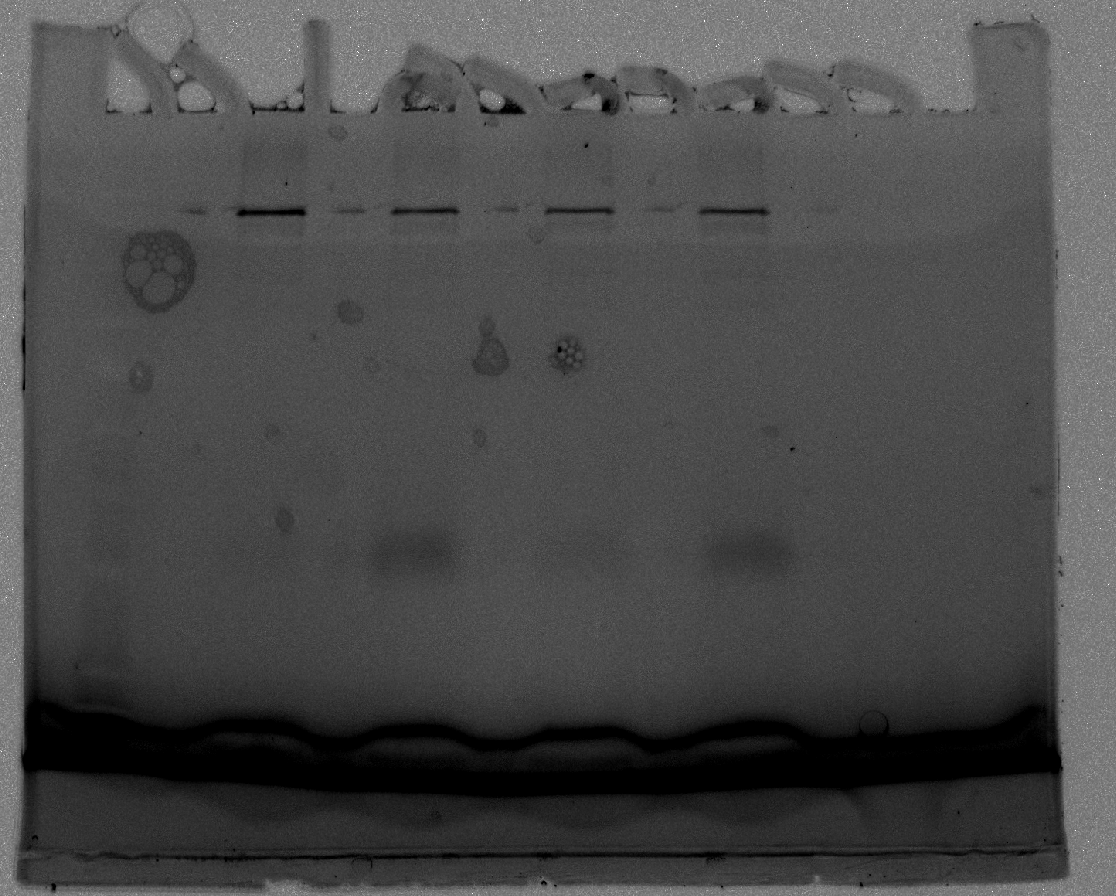

Supplement: Figure 2—source data 2. [file elife-108789-fig2-data2.zip › Figure 2b - source data 2/Fig2b_snap surface 488.tif]

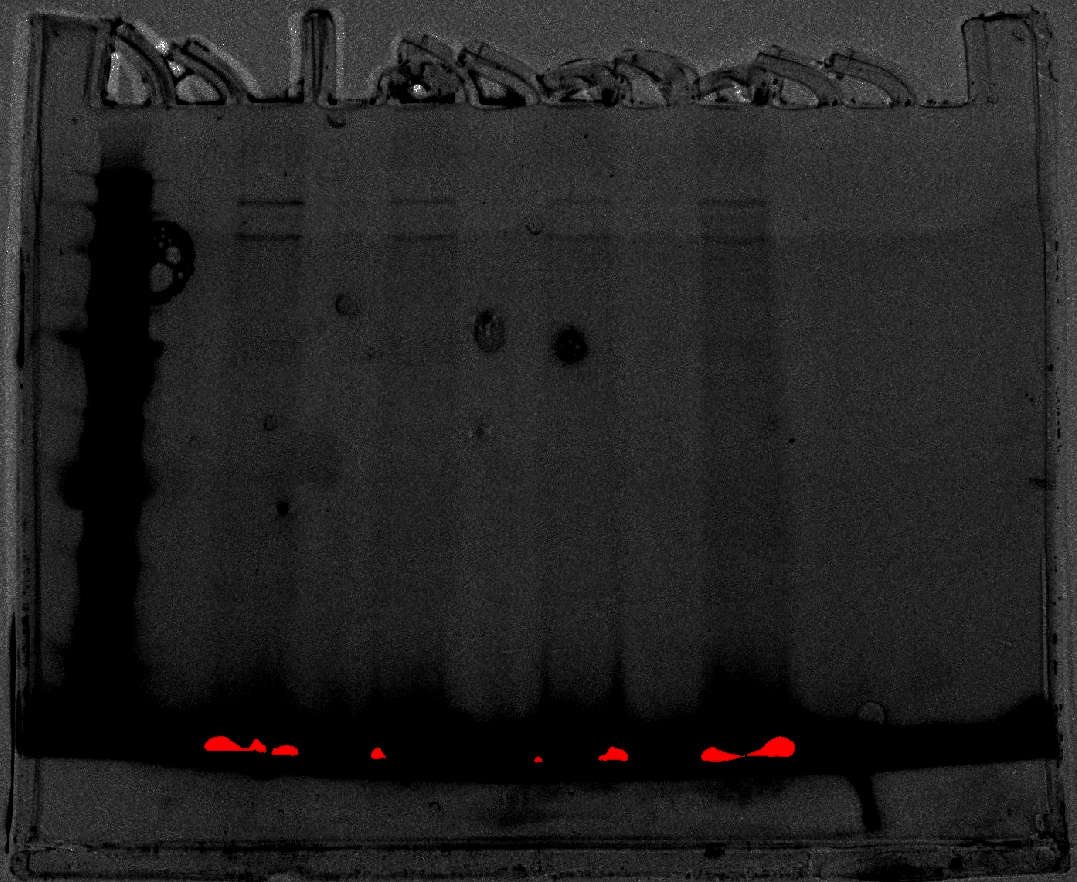

Supplement: Figure 2—source data 2. [file elife-108789-fig2-data2.zip › Figure 2b - source data 2/Figure2b_atto647N.jpg]
