# Supplementary material for: Active regulation of the epidermal growth factor receptor by the membrane bilayer
Source: eLife. 2026 Apr 14;14:RP108789. doi: 10.7554/eLife.108789 (PMC13078784; doi:10.7554/eLife.108789)
Supplement: Figure 1—figure supplement 2—source data 5. [file elife-108789-fig1-figsupp2-data5.pdf]

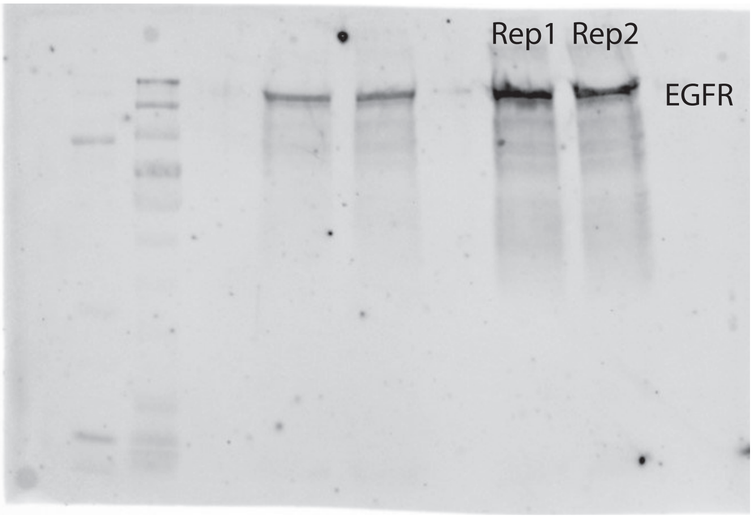


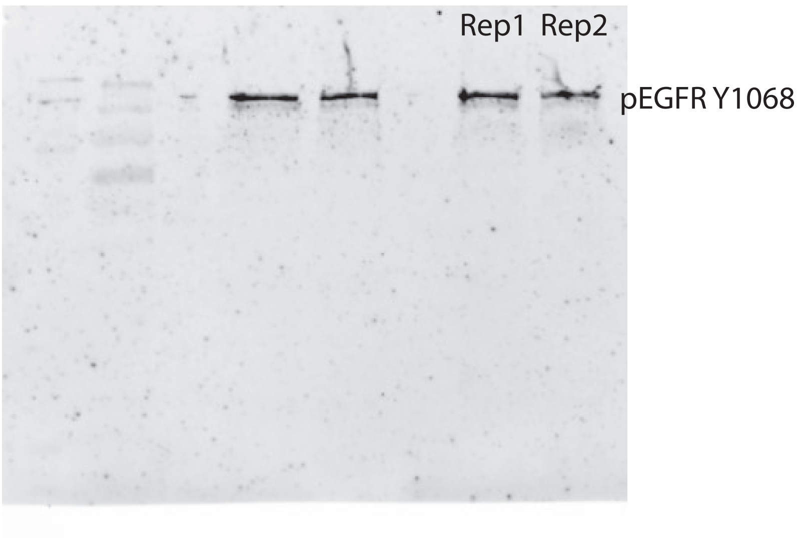


Figure 1, figure supplement 2d, Source Data 1. Original gels corresponding to Figure 1, figure supplement 2d.
