# Supplementary material for: Active regulation of the epidermal growth factor receptor by the membrane bilayer
Source: eLife. 2026 Apr 14;14:RP108789. doi: 10.7554/eLife.108789 (PMC13078784; doi:10.7554/eLife.108789)
Supplement: Figure 1—figure supplement 3—source data 1. [file elife-108789-fig1-figsupp3-data1.zip › Figure_1,_figure_supplement_3_Source_data_1.pdf]

2 hours

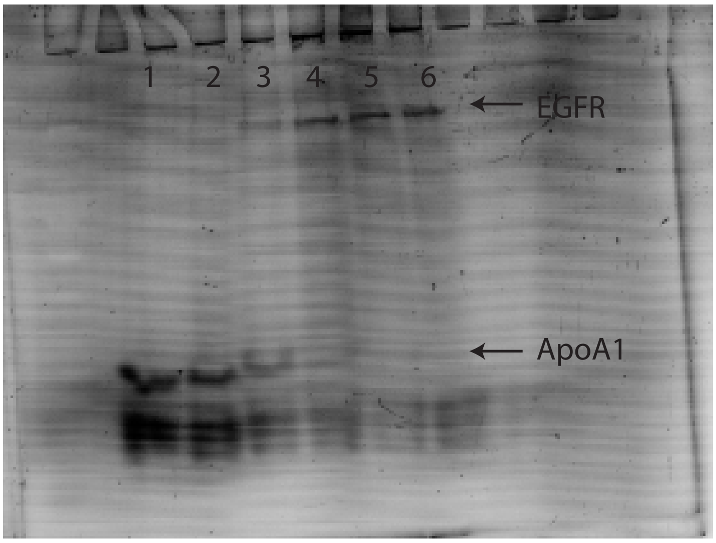

4 hours

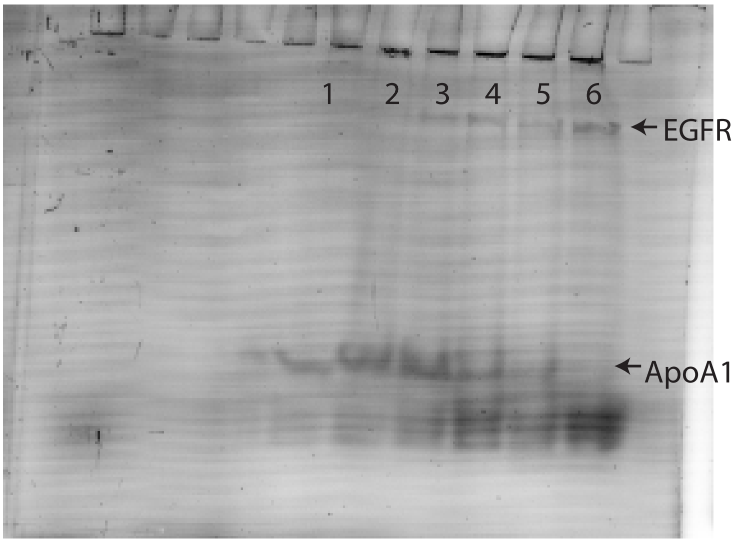

6 hours

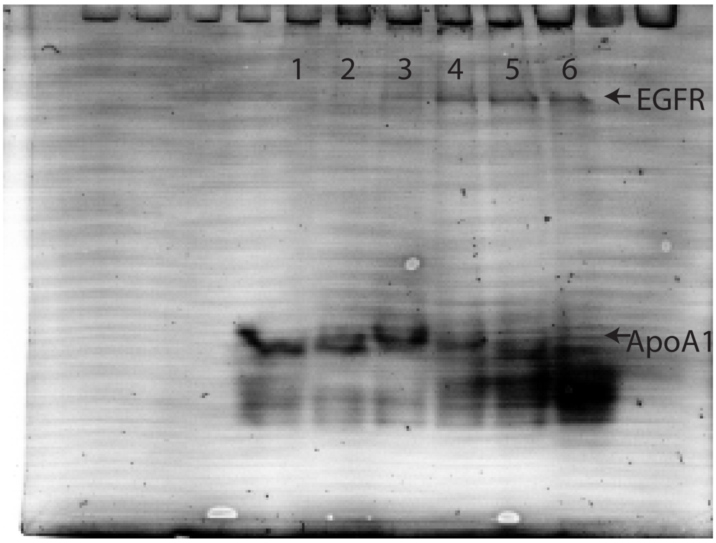

9 hours

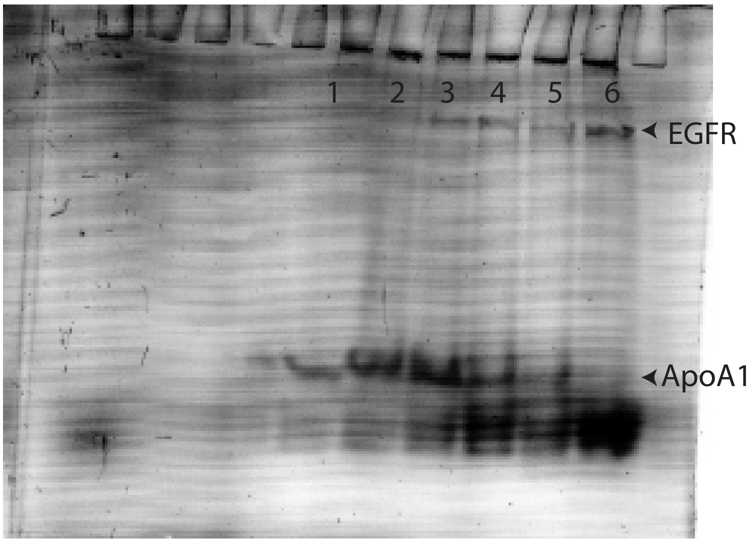

12 hours

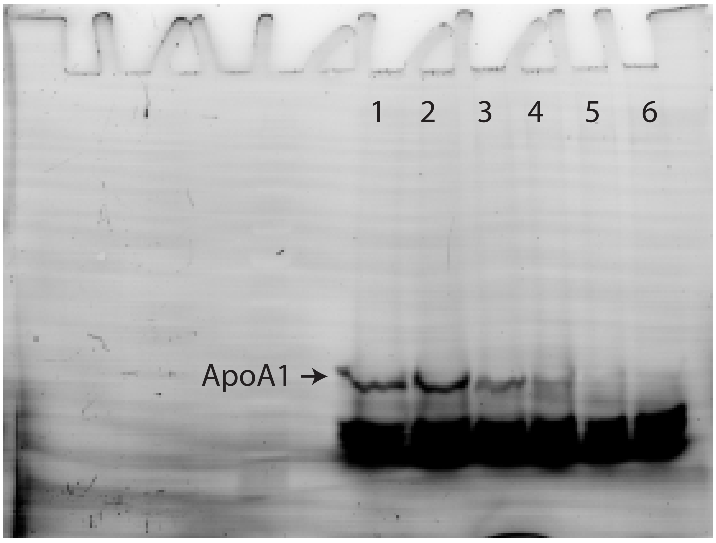

19 hours

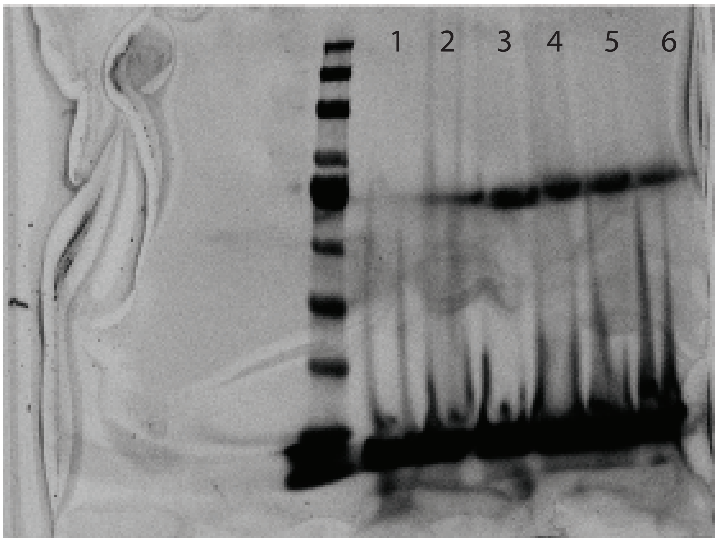

Figure 1, figure supplement 3, Source Data 1. Original gels corresponding to Figure 1, figure supplement 3.
