# Supplementary material for: Active regulation of the epidermal growth factor receptor by the membrane bilayer
Source: eLife. 2026 Apr 14;14:RP108789. doi: 10.7554/eLife.108789 (PMC13078784; doi:10.7554/eLife.108789)
Supplement: Figure 2—source data 1. [file elife-108789-fig2-data1.zip › Figure_2b_Source_data_1.pdf]

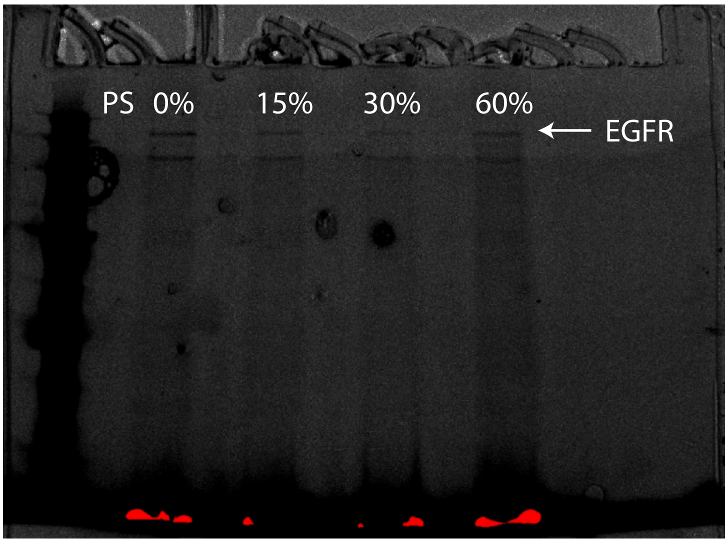

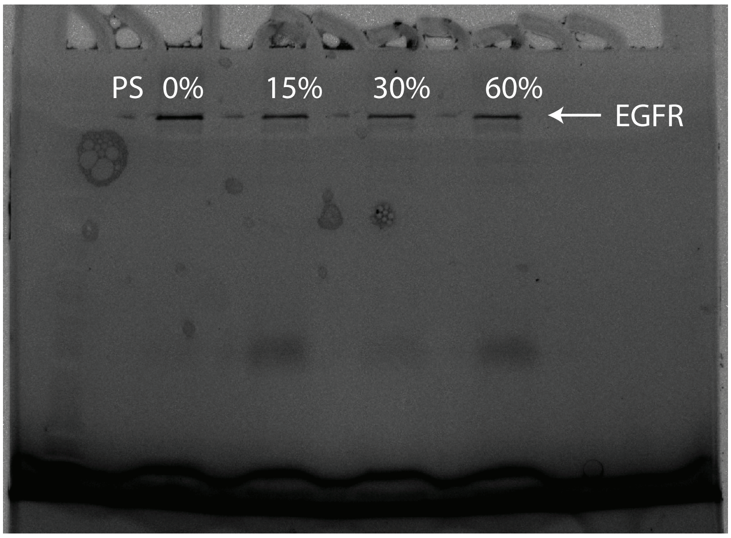

Figure 2b, Source Data 1. Original gels corresponding to Figure2b. (Left) Intensity of atto647N labeled EGFR. (Right) Intensity of snap surface 488 labeled EGFR.
