# Supplementary material for: Active regulation of the epidermal growth factor receptor by the membrane bilayer
Source: eLife. 2026 Apr 14;14:RP108789. doi: 10.7554/eLife.108789 (PMC13078784; doi:10.7554/eLife.108789)
Supplement: Supplementary file 1. [file elife-108789-supp1.pdf]

Sample condition 1	Sample condition 2	P-value	F	Degrees of freedom
0 % anionic lipids				
EGFR, -EGF	EGFR, +EGF	< 0.001	49.4	875
30 % anionic lipids				
EGFR, -EGF	EGFR, +EGF	< 0.001	108.8	863

Table A. Statistical analysis of smFRET lifetime distributions. Results from one-way analysis of variance (ANOVA) with P-value, F-statistic and degrees of freedom for all experimental pairs (experiment 1 and experiment 2 in the above table) from the distributions reported in Fig. 2j, k in the main text.

Sample conditions	Number of molecules	Number of bunches
0 % anionic lipids		
EGFR, -EGF	111	448
EGFR, +EGF	92	428
30 % anionic lipids		
EGFR, -EGF	95	422
EGFR, +EGF	111	442

Table B. Sample sizes for smFRET measurements. The number of molecules and number of photon bunches used to construct the lifetime distributions are reported for all smFRET histograms from Fig. 2j, k in the main text.

Sample condition 1	Sample condition 2	P-value	F	Degrees of freedom
0 % POPS, 100 % DMPC; -EGF	0 % POPS, 100 % DMPC; +EGF	< 0.001	911.3	1398
0 % POPS, 100 % POPC; -EGF	0 % POPS, 100 % POPC; +EGF	< 0.001	204.48	757
15 % POPS, 85 % POPC; -EGF	15 % POPS, 85 % POPC; +EGF	< 0.001	104.87	850
30 % POPS, 70 % POPC; -EGF	30 % POPS, 70 % POPC; +EGF	< 0.001	540.13	1686
60 % POPS, 40 % POPC; -EGF	60 % POPS, 40 % POPC; +EGF	0.7512	0.1	1193

Table C. Statistical analysis of smFRET lifetime distributions. Results from one-way analysis of variance (ANOVA) with P-value, F-statistic and degrees of freedom for all experimental pairs (experiment 1 and experiment 2 in the above table) from the distributions reported in Fig. 3b in the main text and Supplementary Fig. 17.

Sample conditions	Number of molecules	Number of bunches
0 % POPS, 100 % DMPC; -EGF	51	702
0 % POPS, 100 % DMPC; +EGF	53	697
0 % POPS, 100 % DMPC; donor-only	77	992
0 % POPS, 100 % POPC; -EGF	74	422
0 % POPS, 100 % POPC; +EGF	75	336
0 % POPS, 100 % POPC; donor-only	122	1254
15 % POPS, 85 % POPC; -EGF	87	490
15 % POPS, 85 % POPC; +EGF	76	361
15 % POPS, 85 % POPC; donor-only	107	665
30 % POPS, 70 % POPC; -EGF	155	891
30 % POPS, 70 % POPC; +EGF	118	796
30 % POPS, 70 % POPC; donor-only	59	551
60 % POPS, 40 % POPC; -EGF	150	723
60 % POPS, 40 % POPC; +EGF	71	471
60 % POPS, 40 % POPC; donor-only	113	488

Table D. Sample sizes for smFRET measurements. The number of molecules and number of photon bunches used to construct the lifetime distributions are reported for all smFRET histograms from Fig. 3b in the main text and Supplementary Fig. 17.

(a) Two-Gaussian model:	
$\mu 1$	1.33 ns
$\sigma 1$	0.34 ns
$\mu 2$	2.71 ns
$\sigma 2$	0.67 ns

(b) Three-Gaussian model:	
$\mu 1$	1.38 ns
$\sigma 1$	0.36 ns
$\mu 2$	2.64 ns
$\sigma 2$	0.45 ns
$\mu 3$	3.43 ns
$\sigma 3$	0.64 ns

(c) BIC analysis and Ashman's D analysis for two-gaussian model:	
Likelihood	-10468.93
BIC	20983.64
D12	2.60

(d) BIC analysis and Ashman's D analysis for three-gaussian model:	
Likelihood	-10190.78
BIC	20454.82
D12	3.09
D13	3.95
D23	1.43

Table E. Supplementary Table 1. Model selection and statistical analysis of global fits. Comparison of two- and three-Gaussian models used to globally fit the lifetime distributions across all experimental conditions. Parameters (μ, σ) correspond to the mean and standard deviation of each Gaussian component, respectively. Likelihood, Bayesian Information Criterion (BIC), and Ashman's D values are reported for assessing model quality and separation of components. The number of free parameters is 5 for the two-Gaussian model and 8 for the three-Gaussian model. The number of single-molecule photon bunches = 9,483 across all 18 conditions (lipid composition and $\pm$EGF).

Sample conditions	Compact state	Open state
0 % POPS, 100 % DMPC; -EGF	5.4 % [3.8 %, 6.9 %]	95 % [93 %, 96 %]
0 % POPS, 100 % DMPC; +EGF	58 % [54 %, 62 %]	42 % [38 %, 46 %]
0 % POPS, 100 % POPC; -EGF	13 % [10 %, 16 %]	87 % [84 %, 90 %]
0 % POPS, 100 % POPC; +EGF	40 % [34 %, 46 %]	60 % [54 %, 66 %]
15 % POPS, 85 % POPC; -EGF	31 % [25%, 37 %]	69 % [63 %, 75 %]
15 % POPS, 85 % POPC; +EGF	8.8 % [8.7 %, 9.0 %]	91 % [91 %, 91 %]
30 % POPS, 70 % POPC; -EGF	26 % [19 %, 33 %]	74 % [67 %, 81 %]
30 % POPS, 70 % POPC; +EGF	4.0 % [3.4 %, 4.6 %]	96 % [95 %, 97 %]
60 % POPS, 40 % POPC; -EGF	6.3 % [5.2 %, 7.4 %]	94 % [93 %, 95 %]
60 % POPS, 40 % POPC; +EGF	2.1 % [1.2 %, 2.9 %]	98 % [97 %, 99 %]

Table F. Amplitude of compact and open state of the EGFR intracellular domain from smFRET lifetime distributions in main text Fig. 3b. The numbers in parenthesis indicates the error bar indicated in Fig. 3g.

Sample condition 1	Sample condition 2	P-value	F	Degrees of freedom
92.5 % POPC, 7.5 % Cholesterol; -EGF	92.5 % POPC, 7.5 % Cholesterol; +EGF	0.91	0.01	975
80 % POPC, 20 % Cholesterol; -EGF	80 % POPC, 20 % Cholesterol; +EGF	0.8245	0.05	600

Table G. Statistical analysis of smFRET lifetime distributions. Results from one-way analysis of variance (ANOVA) with P-value, F-statistic and degrees of freedom for all experimental pairs (experiment 1 and experiment 2 in the above table) from the distributions reported in Fig. 3d in the main text.

Sample conditions	Number of molecules	Number of bunches
92.5 % POPC, 7.5 % Cholesterol; -EGF	71	596
92.5 % POPC, 7.5 % Cholesterol; +EGF	59	380
92.5 % POPC, 7.5 % Cholesterol; donor-only	58	1255
80 % POPC, 20 % Cholesterol; -EGF	62	315
80 % POPC, 20 % Cholesterol; +EGF	58	286
80 % POPC, 20 % Cholesterol; donor-only	48	601

Table H. Sample sizes for smFRET measurements. The number of molecules and number of photon bunches used to construct the lifetime distributions are reported for all smFRET histograms from Fig. 3d in the main text.

Sample conditions	Compact state	Open state
92.5 % POPC,<br>7.5 % Cholesterol; -EGF	11 % [8.7 %, 13 %]	89 % [87 %, 91 %]
92.5 % POPC,<br>7.5 % Cholesterol; +EGF	10 % [7.2 %, 12 %]	90 % [88 %, 93 %]
80 % POPC,<br>20 % Cholesterol; -EGF	4.6 % [3.9 %, 5.4 %]	95 % [95 %, 96 %]
80 % POPC,<br>20 % Cholesterol; +EGF	6.0 % [5.1 %, 6.9 %]	94 % [93 %, 95 %]

Table I. Amplitude of compact and open state of the EGFR intracellular domain from smFRET lifetime distributions in main text Fig. 3d. The numbers in parenthesis indicates the error bar indicated in Fig. 3g.

Sample condition 1	Sample condition 2	P-value	F	Degrees of freedom
62.5 % POPC, 30 % POPS, 7.5 % Cholesterol, -EGF	62.5 % POPC, 30 % POPS, 7.5 % Cholesterol, +EGF	0.61	0.27	1203
50 % POPC, 30 % POPS, 20 % Cholesterol, -EGF	50 % POPC, 30 % POPS, 20 % Cholesterol, +EGF	0.002	9.88	794

Table J. Statistical analysis of smFRET lifetime distributions. Results from one-way analysis of variance (ANOVA) with P-value, F-statistic and degrees of freedom for all experimental pairs (experiment 1 and experiment 2 in the above table) from the distributions reported in Fig. 3f in the main text.

Sample conditions	Number of molecules	Number of bunches
62.5 % POPC, 30 % POPS, 7.5 % Cholesterol; -EGF	42	489
62.5 % POPC, 30 % POPS, 7.5 % Cholesterol; +EGF	55	715
62.5 % POPC, 30 % POPS, 7.5 % Cholesterol; donor-only	75	783
50 % POPC, 30 % POPS, 20 % Cholesterol; -EGF	81	390
50 % POPC, 30 % POPS, 20 % Cholesterol; +EGF	71	405
50 % POPC, 30 % POPS, 20 % Cholesterol; donor-only	66	877

Table K. Sample sizes for smFRET measurements. The number of molecules and number of photon bunches used to construct the lifetime distributions are reported for all smFRET histograms from Fig. 3f in the main text.

Sample conditions	Compact state	Open state
62.5 % POPC, 30 % POPS, 7.5 % Cholesterol, -EGF	0.0 % [0.0 %, 0.0 %]	100 % [100 %, 100 %]
62.5 % POPC, 30 % POPS, 7.5 % Cholesterol, +EGF	5.9 % [4.8 %, 7.0 %]	94 % [93 %, 95 %]
50 % POPC, 30 % POPS, 20 % Cholesterol, -EGF	0 % [0 %, 0 %]	100 % [100 %, 100 %]
50 % POPC, 30 % POPS, 20 % Cholesterol, +EGF	0.9 % [0.8 %, 1.0 %]	99 % [99 %, 99 %]

Table L. Amplitude of compact and open state of the EGFR intracellular domain from smFRET lifetime distributions in main text Fig. 3f. The numbers in parenthesis indicates the error bar indicated in Fig. 3g.

Antibody name	Target	Host	Company	Clone	Dilution
Anti-EGFR Antibody (A-10)	EGFR C-terminal	MouseIgG$_{2a}$	Santa Cruz Biotechnology	Monoclonal	1:200
Human Phospho-EGFR Y1068 Antibody	EGFR phosphorylated at Y1068	MouseIgG$_{2a}$	R&D Systems	Monoclonal	1:200
Goat anti-Mouse IgG (H+L) Highly Cross-Adsorbed Antibody Alexa Fluor 790	Mouse	GoatIgG	Thermo Fisher	Polyclonal	1:10000

Table M. List of antibodies used to show phosphorylation of EGFR nanodiscs.
